# Sleep Disturbances and Health-Related Quality of Life in Paediatric Oncology: A Comparative Study with Non-Oncological Chronic Illness and Healthy Peers

**DOI:** 10.3390/diseases14100356

**Published:** 2026-09-26

**Authors:** Shuk Yan Mak, Jeanny Sui Sum Cheung, Ankie Tan Cheung, William Ho Cheung Li

**Affiliations:** 1 Paediatric Haematology and Oncology Centre, Department of Paediatrics and Adolescent Medicine, Hong Kong Children’s Hospital, Hong Kong SAR, China; css213@ha.org.hk; 2 The Nethersole School of Nursing, The Chinese University of Hong Kong, Hong Kong SAR, China; ankiecheung@cuhk.edu.hk (A.T.C.); williamli@cuhk.edu.hk (W.H.C.L.)

**Keywords:** paediatric oncology, sleep disturbance, sleep quality, health-related quality of life, symptom burden, supportive care, chronic illness, patient-reported outcomes

## Abstract

**Background/Objectives:** Sleep disturbance is increasingly recognized as a key contributor to symptom burden and functioning in paediatric oncology, yet comparative evidence across clinical and healthy populations remains limited. This study examined sleep quality and its association with health-related quality of life (HRQoL) in children and adolescents with cancer compared with peers with non-oncological chronic illnesses and healthy controls. **Methods**: A cross-sectional study was conducted at Hong Kong Children’s Hospital. Participants 6–18 years were recruited by convenience sampling into three groups (oncology, chronic illness, healthy; *n* = 50 each). Children and adolescents completed the Chinese Pittsburgh Sleep Quality Index (PSQI) and Pediatrics Quality of Life Inventory (PedsQL) 4.0. Group differences were tested using chi-square tests and one-way analysis of variance (ANOVA) with post hoc Tukey comparisons. Pearson correlations and hierarchical multiple linear regression examined PSQI-PedsQL associations. **Results**: Sleep quality differed significantly across groups (*p* < 0.001), with the poorest sleep observed in the oncology group. The prevalence of poor sleep (PSQI ≥ 5) was 60.0% in oncology group, compared with 40.0% in those with chronic illness and 30.0% in healthy peers. Mean global PSQI scores were 6.02 ± 3.40 in the oncology group, 4.10 ± 2.60 in the chronic illness group, and 3.30 ± 2.35 in healthy controls, with a significant overall group difference (*p* < 0.001). Impairments were most pronounced in sleep latency, habitual sleep efficiency, and sleep disturbances. HRQoL was lowest in the oncology group (*p* < 0.001). Mean PedsQL total scores were 68.98 ± 15.73 in the oncology group, 75.99 ± 14.03 in the chronic illness group, and 85.44 ± 10.26 in healthy controls, respectively, with a significant overall group difference (*p* < 0.001). Across the full sample, poor sleep quality was independently associated with lower HRQoL after adjustment for demographic and clinical factors (β = −0.271, *p* = 0.002), with no significant interaction by group. **Conclusions**: Children and adolescents with cancer reported clinically significant sleep impairment, driven mainly by reduced sleep quality and continuity. Routine sleep screening and targeted supportive interventions may improve HRQoL in paediatric oncology.

## 1. Introduction

Sleep is a fundamental biological process that supports neurocognitive development, immune and endocrine function, emotional regulation, and learning in children and adolescents [1,2]. Disrupted sleep in paediatric populations is associated with impaired attention, mood problems, lower academic performance, and reduced quality of life [3,4,5]. Sleep is increasingly recognized in clinical paediatrics as both an outcome and a potentially modifiable factor associated with symptom burden and functional recovery.

Children and adolescents with cancer face an elevated risk of sleep disturbance related to disease symptoms (e.g., pain, nausea), with the prevalence of sleep disturbances in Western countries ranging from 40% to 80% driven by treatment side effects (e.g., corticosteroid-associated insomnia), anxiety, and disruptions to routines during hospitalizations [6,7,8,9,10]. Sleep problems in paediatric oncology have been reported across the treatment trajectory, including during active therapy and survivorship. They can manifest as prolonged sleep latency, increased nocturnal awakenings, and heightened daytime sleepiness or fatigue [11]. Fatigue and sleep disruption can reinforce each other, perpetuating circadian misalignment. Despite this clinical importance, sleep concerns in paediatric oncology are often under-assessed and are rarely integrated as routine supportive care [12]. A recent systematic review yielded heterogeneous findings across psychoeducational, behavioral, physical activity, pharmacotherapy, and massage approaches, underscoring the paucity of large-scale randomized controlled trials and the absence of established clinical guidelines [13].

The clinical implications of sleep disruption in children and adolescents with cancer are beyond nighttime distress. Inadequate sleep among children and adolescents with cancer can increase pain sensitivity, aggravate fatigue levels, and exacerbate mood difficulties, potentially reducing treatment adherence and engagement in rehabilitation or education [8,11,13]. These sleep-related sequelae align with broader health-related quality of life (HRQoL) impacts, as poor sleep has been associated with greater emotional distress, reduced coping, and lower participation in school and social activities [14,15,16,17]. Sleep disturbances are also prevalent in paediatric chronic illness populations and have been linked to poorer physical functioning, increased daytime impairment, and lower psychosocial well-being, indicating that sleep may represent a common, clinically relevant factor associated with daily functioning in children and adolescents with chronic health conditions [18,19,20,21,22]. In contrast, healthy children and adolescents typically exhibit stable sleep patterns, providing a benchmark for illness-related disruption. However, the extent to which sleep mediates differences across illness groups remains uncertain. Caregivers can also be affected by children’s nighttime distress, with consequences for family functioning and caregiving capacity [23,24]. Sleep quality is not merely a secondary comfort issue but a critical determinant that intersects with multiple symptom clusters and functional outcomes relevant to patients, caregivers, and clinical teams.

A growing body of evidence indicates that sleep disturbance is highly prevalent among paediatric oncology children and adolescents, with research suggesting that over half of these children and adolescents experience significant disruptions across various stages of diagnosis and treatment [10,11], yet the availability of routine screening and non-pharmacological sleep support varies widely across health systems and care settings [13]. In Hong Kong, evidence on sleep quality in paediatric oncology and its association with HRQoL remains limited. Local evidence is needed as cultural practices, housing conditions, school schedules, and healthcare systems can shape sleep routines and perceptions, underscoring the need for locally grounded research to inform culturally sensitive interventions and policy development. Moreover, routine oncology care often lacks structured pathways for sleep assessment, education on sleep hygiene, or behavioral intervention, leading to normalization of sleep problems, delayed attention until symptoms become severe, and overreliance on pharmacological approaches without addressing behavioral and environmental contributors [12].

A comparative research approach is warranted given the identified gaps. Such designs can delineate sleep disruptions that are common across chronic paediatric conditions from those that may be uniquely pronounced in oncology, including treatment-specific effects and recurrent inpatient disruptions. Many paediatric disorders can also alter sleep through mechanisms such as pain, respiratory symptoms, medication side effects, or psychological stress. Without appropriate comparison groups, it remains difficult to determine whether the sleep burden in paediatric oncology is distinct in severity or pattern. This distinction is clinically significant, as it informs prioritization of supportive care. If children and adolescents with cancer demonstrate a higher prevalence of poor sleep or worse component scores relative to other chronic illness groups, greater emphasis on systematic sleep assessment and targeted intervention should be warranted within oncological supportive care.

## 2. Materials and Methods

### 2.1. Study Aim

This was a cross-sectional study conducted at Hong Kong Children’s Hospital (HKCH) that examined sleep quality in paediatric oncology children and adolescents relative to healthy peers and children and adolescents with non-oncological chronic illnesses. The specific objectives of the study are to: (1) estimate the prevalence of poor sleep, defined by thePittsburgh Sleep Quality Index (PSQI) global score ≥5 and to characterize PSQI component profiles among children with cancer in Hong Kong; (2) compare PSQI global and component scores across paediatric oncology children and adolescents, children and adolescents with non-oncological chronic illnesses, and healthy peers using appropriate statistical tests with post hoc analyses; and (3) examine associations between sleep quality and HRQoL. By quantifying group differences and HRQoL associations, this study aims to provide evidence that sleep is a clinically meaningful and potentially modifiable target in paediatric oncology supportive care, while providing a foundation for future service development and intervention research in Hong Kong.

### 2.2. Study Design and Participants

A cross-sectional study was conducted at HKCH from July to November 2025. Children and adolescents aged 6–18 years were recruited by convenience sampling into three predefined groups according to clinical or health status: oncology non-oncological chronic illnesses, and healthy controls. The pragmatic recruitment target was 150 participants, with 50 per group, based on study feasibility and the anticipated availability of eligible participants during the recruitment period. No formal a priori sample size calculation was performed. A total of 150 individuals were assessed for eligibility, with 50 in each group; all met the eligibility criteria and were invited to participate. No additional individuals were screened. All 150 enrolled participants completed the study questionnaires and were included in the final analysis. No participants were excluded from the final analysis. Participant flow is presented in Figure 1.

The oncology group comprised children and adolescents with a confirmed cancer diagnosis who were hospitalized in inpatient oncology wards and actively receiving cancer-directed treatment, such as chemotherapy or radiotherapy. The non-oncological chronic illness group comprised children and adolescents hospitalized for non-oncological chronic conditions requiring ongoing medical care (e.g., asthma, type 1 diabetes, or chronic kidney disease), recruited from the inpatient cardiothoracic, renal, and general paediatrics units at HKCH. For the purpose of this study, chronic illness was operationally defined as a long-term health condition expected to last for one year or more and requiring continuing medical management. No minimum duration of illness before recruitment was required, as the included diagnoses were expected to persist beyond one year from diagnosis. Conditions represented in the enrolled sample included renal, cardiac, haematological/immunological, and neurometabolic conditions. For the oncology and non-oncological chronic illness groups, the researcher approached potentially eligible inpatients and their families in the relevant hospital units to invite participation.

Healthy controls were children and adolescents recruited from children of hospital staff and students at a secondary school. The researcher directly approached hospital staff families to invite participation. For school-based recruitment, the researcher first contacted a teacher and subsequently distributed study questionnaires and consent forms to students and their families. Eligibility required an age of 6–18 years and no known chronic medical, psychological, developmental, or diagnosed sleep conditions based on parent- or guardian-reported screening. The groups were not individually matched. However, recruitment was monitored to obtain broadly comparable age and sex distributions across the three groups. The final sample showed no statistically significant differences in age or sex across groups. Age and sex were also included as covariates in the multivariable regression analysis.

Exclusion criteria for all groups included severe neurocognitive impairment, acute critical illness, inability to complete questionnaires in Chinese, and a known primary sleep disorder or other diagnosed sleep condition. For the oncology and chronic illness groups, eligibility and exclusion criteria were verified through medical records where available. For healthy controls, health status and absence of known chronic illness or diagnosed sleep condition were confirmed using parent- or guardian-reported screening information. Ethical approval was obtained from the HKCH Research Ethics Committee (Reference number: PAED-2025-045). Written informed consent from parents or legal guardians was obtained for all participants in accordance with institutional ethics requirements. Child assent was obtained from participants aged 6–12 years where required by the institution before participation.

### 2.3. Data Collection

Data were collected using self-report questionnaires. Participants in the oncology and chronic illness groups completed the questionnaires during their inpatient stay, whereas healthy controls completed them in a community setting, primarily at home. The same standardized Chinese-language questionnaires and administration procedures were used across all groups. All participants completed standardized and validated Chinese-language instruments. Older children and adolescents completed the questionnaires independently whenever possible. For younger children or participants with reading or comprehension difficulties, parents, guardians, or research staff could provide practical assistance, such as reading items aloud or clarifying response options. However, they were instructed not to answer on behalf of the child. The Chinese version of the PSQI is a 19-item self-report measure that assesses sleep quality over the past month across seven distinct components: subjective sleep quality, sleep latency, sleep duration, habitual sleep efficiency, sleep disturbances, sleep medication use, and daytime dysfunction [25]. Each of the seven PSQI components is scored from 0 to 3 and summed to produce a global score ranging from 0 to 21, with higher scores indicating poorer sleep quality. A global PSQI score ≥ 5 was used as a screening threshold for poor sleep quality rather than as a diagnostic criterion [26,27]. The Chinese-language PSQI has previously been used in paediatric oncology-related research, including studies involving childhood cancer survivors, in which acceptable psychometric properties have been reported [28]. In the present study, the PSQI was used as a generic screening measure of subjective sleep quality to enable standardized comparisons across study groups. Although the PSQI was originally developed for adults, it has been used in studies involving school-aged children and adolescents, including children with chronic health conditions and childhood cancer survivors [29]. In the present study, the PSQI was used as a screening measure of subjective sleep quality rather than as a diagnostic instrument. Routine nursing documentation did not routinely include a standardized assessment of sleep quality; therefore, sleep outcomes were assessed using the validated Chinese version of the Pittsburgh Sleep Quality Index (PSQI).

HRQoL was measured using the Chinese version of the Pediatrics Quality of Life Inventory (PedsQL) 4.0, a 23-item instrument encompassing four domains: physical, emotional, social, and school functioning. Item scores are transformed to 0–100 scale, with higher scores indicating better HRQoL. The child self-report version was used for all participants; younger children could receive assistance with reading or understanding questions, but responses reflected the child’s own answers. The Chinese PedsQL 4.0 has undergone cross-cultural adaptation and validation, demonstrating high internal consistency (Cronbach’s alpha > 0.80) and strong construct validity, making it a reliable and appropriate tool for evaluating HRQoL in Chinese paediatric populations [30,31].

Demographic information, including age, sex, and parental education, was obtained from parent- or guardian-completed questionnaires. Clinical information for participants in the oncology and chronic illness groups, including disease type, relapse status, and hypnotic use, was extracted from medical records. For healthy controls, eligibility and health status were based on parent- or guardian-reported screening information.

### 2.4. Statistical Analysis

Data were analyzed using SPSS version 28.0. Descriptive statistics were used to summarize participant characteristics. Normality was assessed using histograms and normal probability (Q-Q) plots, and formal normality tests where appropriate. Homogeneity of variances was evaluated using Levene’s test, and linearity was assessed using scatterplots prior to correlation analyses.

Although the groups were not individually matched, recruitment was monitored to achieve broadly comparable age and sex distributions across groups. Age and sex were also included as covariates in the multiple linear regression analysis to account for their potential influence on HRQoL.

Categorical variables were analyzed using chi-square tests or Fisher’s exact tests, as appropriate. For continuous outcomes, including PSQI global score, PedsQL total score, and PedsQL domain scores, group differences were assessed using one-way analysis of variance (ANOVA) with Tukey’s honestly significant difference (HSD) post hoc tests when assumptions were considered acceptable. Because the PSQI component scores are ordinal and bounded, Kruskal–Wallis tests were additionally conducted as non-parametric sensitivity analyses for the PSQI global score and component scores (Supplementary Tables S1 and S2). These sensitivity analyses were used to evaluate whether the interpretation of the ANOVA-based group comparisons was stable.

Associations between sleep quality and HRQoL were examined using Pearson correlation coefficients when assumptions of normality and linearity were met; otherwise, Spearman’s rank correlation coefficients were used. Multiple linear regression analysis was conducted to determine whether PSQI global score predicted PedsQL total score after adjusting for group, age, gender, parental education, and hypnotic use. Prior to conducting the regression analyses, model assumptions were assessed. Linearity and homoscedasticity were evaluated using residual scatterplots, normality of residuals was assessed using histograms and normal probability (Q-Q) plots, independence of errors was examined using the Durbin-Watson statistic, and multicollinearity was assessed using variance inflation factor (VIF) values. Potentially influential observations were evaluated using standardized residuals and Cook’s distance values. All assumptions were considered satisfactory before interpretation of the regression models. Interaction terms (PSQI × group) were included to assess whether the association between sleep quality and HRQoL differed across study groups. Post hoc power analyses were conducted based on the final sample size and observed effect sizes for the main group comparisons and regression models. For the moderation analysis, Cohen’s f^2^ was calculated from the change in R^2^ after adding the interaction terms.

## 3. Results

Participants’ characteristics are presented in Table 1. Mean age did not differ significantly across groups, and the sex distribution was comparable. Parents’ education varied by group, with a higher proportion of tertiary education in the healthy group. Hypnotic use was reported exclusively in the oncology group (10%).

### 3.1. Sleep Quality

Global PSQI scores differed significantly across groups (Table 2), with the oncology group reporting the poorest sleep quality, followed by the chronic illness group, and the healthy group reporting the lowest scores. Overall, 43.3% (65/150) of participants were classified as poor sleepers (PSQI ≥ 5). The prevalence of poor sleep was highest in the oncology group (60.0%), compared with the chronic illness group (40.0%) and the healthy group (30.0%).

### 3.2. PSQI Component Scores

Significant group differences were observed across several PSQI components. The oncology group reported poorer subjective sleep quality, longer sleep latency, lower habitual sleep efficiency, and greater sleep disturbances than both comparison groups. Hypnotic use was reported only in the oncology group. Sleep duration did not differ significantly across groups, and differences in daytime dysfunction were not statistically significant, although scores were numerically higher in the oncology group.

Symptom-level analyses supported these component findings and showed that elevated PSQI burden in paediatric oncology reflected specific disruptions. Children and adolescents with cancer were more frequently reported to take longer than 30 min to fall asleep (*p* = 0.0018) and experience nocturnal or early morning awakenings (*p* = 0.0089). Somatic sleep-related complaints showed the strongest group effects, particularly nocturia (*p* < 0.0001), with the highest prevalence in the oncology group, followed by the chronic illness and healthy groups. Thermoregulatory discomfort (feeling too cold, *p* = 0.00043; feeling too hot, *p* = 0.0092) and pain-related sleep disturbance (*p* = 0.00049) were also more frequently reported in the oncology group. In contrast, breathing discomfort (*p* = 0.225), coughing or loud snoring (*p* = 0.514), and bad dreams (*p* = 0.896) did not differ between groups. Overall, the sleep profile in the oncology group was characterised by greater difficulties initiating and maintaining sleep, accompanied by increased nocturnal discomfort, rather than reduced sleep duration.

### 3.3. Associations with Demographic and Clinical Factors

Across the total sample, age was weakly positively correlated with global PSQI score (r = 0.169, *p* = 0.038). In stratified analyses, this association was significant only in the healthy group (r = 0.517, *p* < 0.001), but not in the oncology or chronic illness groups. PSQI scores did not differ by sex (t(148) = −1.767, *p* = 0.079). There were no significant differences in PSQI scores by oncology subtype (F(1,48) = 0.002, *p* = 0.963) or chronic illness subtype (F(4,45) = 0.699, *p* = 0.597).

### 3.4. Health-Related Quality of Life

PedsQL total scores differed significantly across groups (Table 3). The healthy group reported the highest HRQoL, followed by the chronic illness group, with the oncology group reporting the lowest scores. Post hoc analyses indicated that all pairwise group differences were statistically significant.

For PedsQL domains, significant group differences were observed in physical, emotional, and school functioning, as well as in the psychosocial health summary score, with a consistent gradient (healthy > chronic illness > oncology). Social functioning did not differ significantly across groups.

### 3.5. Associations Between Sleep Quality and HRQoL

Across the full sample, poorer sleep quality (higher PSQI scores) was significantly associated with lower HRQoL (lower PedsQL total scores) (Table 4). In stratified analyses, this association reached statistical significance only in the healthy group and not in the oncology or chronic illness groups.

### 3.6. Multivariable Analyses

Regression diagnostics indicated no substantial violations of model assumptions. In the final interaction model, variance inflation factor values ranged from 1.106 to 5.709, tolerance values ranged from 0.175 to 0.904, and the Durbin–Watson statistic was 2.087. Standardized residuals ranged from −2.516 to 1.929, and the maximum Cook’s distance was 0.203, indicating no influential outlying observations.

Before creating interaction terms, global Pittsburgh Sleep Quality Index scores were mean-centered by subtracting the total sample mean score from each participant’s observed score. Interaction terms were then created by multiplying the mean-centered Pittsburgh Sleep Quality Index variable by the group dummy variables, with the healthy group used as the reference group.

Hierarchical multiple linear regression was performed with PedsQL total score as the dependent variable. Model 1 included global PSQI score, study group, age, sex, parental education, and hypnotic use (Table 5). Model 2 additionally included PSQI × group interaction terms (Table 6).

In Model 1, after adjusting for group, age, sex, parental education, and hypnotic use, poorer sleep quality remained a significant predictor of lower PedsQL total scores (B = −1.347, SE = 0.436, β = −0.271, *p* = 0.002). Compared with the healthy group, both the oncology group (B = −13.51, SE = 2.935, β = −0.425, *p* < 0.001) and the chronic illness group (B = −8.485, SE = 2.772, β = −0.267, *p* = 0.003) had significantly lower HRQoL.

In Model 2, higher PSQI scores were associated with lower PedsQL total scores in the healthy reference group (B = −2.246, SE = 0.860, β = −0.452, *p* = 0.010). Neither interaction term was statistically significant, and the interaction block did not significantly improve model fit (ΔR^2^ = 0.009, ΔF(2,138) = 0.829, *p* = 0.438). Thus, there was insufficient evidence that the PSQI–PedsQL association differed across groups.

### 3.7. Post Hoc Power Analysis

A post hoc power analysis based on the observed effect sizes was conducted to evaluate whether the available sample size was adequate for the main analyses. The sample size of 150 participants provided high achieved power for detecting the observed group differences in global Pittsburgh Sleep Quality Index scores and global Pediatric Quality of Life Inventory scores. The achieved power for the full regression model was also high. In Model 2, the full regression model was significant, R^2^ = 0.277, adjusted R^2^ = 0.219, F(11,138) = 4.808, *p* < 0.001. However, the interaction block showed a very small effect size, ΔR^2^ = 0.009, F-change(2, 138) = 0.829, *p* = 0.438, corresponding to Cohen’s f^2^ = 0.012 and achieved power of approximately 0.20. Therefore, the non-significant interaction findings should be interpreted cautiously.

## 4. Discussion

This study provides novel comparative evidence on sleep quality among children and adolescents with cancer, children and adolescents with other chronic illnesses, and healthy children and adolescents within a Chinese population. The findings indicate a substantial burden of sleep disturbance in paediatric oncology and demonstrate its association with poorer HRQoL, supporting the growing recognition of sleep as a clinically relevant component of supportive care.

The prevalence of poor sleep observed in the oncology group (60%) is consistent with prior reports [6,7,8,10,11], indicating that sleep disturbances affect a substantial proportion of children and adolescents undergoing cancer treatment. Importantly, the comparative design of the present study extends previous work by showing that sleep problems are not only common but also more pronounced in paediatric oncology than in both healthy peers and other chronic illness groups. This suggests that cancer-related factors, including treatment intensity, symptom burden, and hospital-related disruptions, may confer additional risk beyond that associated with chronic illness more broadly.

At the component level, sleep impairment in the oncology group was characterised by prolonged sleep latency, reduced habitual sleep efficiency, and increased sleep disturbances, whereas sleep duration did not differ significantly across groups. This pattern aligns with previous studies describing difficulties in sleep initiation and maintenance in paediatric oncology populations [6,7,8,32]. However, inconsistent with some earlier findings reporting reduced sleep duration [6,8,11], the current results suggest that sleep disruption in this group may be driven more by fragmentation and perceived sleep quality than by reduced total sleep time. This distinction is clinically relevant, as poor sleep continuity has been linked to daytime dysfunction independent of sleep duration.

Symptom-level findings further support this interpretation. Children and adolescents in the oncology group more frequently reported difficulty falling asleep, nocturnal awakenings, nocturia, thermal discomfort, and pain interfering with sleep. These findings are consistent with previous literature showing that sleep disruption in paediatric oncology is often accompanied by multiple nocturnal symptoms and environmental challenges during treatment and hospitalization [6,8,33,34,35,36]. However, because detailed symptom severity and treatment-related variables, such as anaemia, nausea and vomiting, corticosteroid exposure, mucositis, anorexia, and pain severity, were not systematically collected in the present study, it was not possible to determine whether these sleep disturbances were primarily disease-related or treatment-related. Therefore, the present findings should be interpreted as describing the pattern of sleep disruption experienced by children and adolescents with cancer rather than identifying its underlying causes. Nevertheless, the prominence of difficulties initiating and maintaining sleep, together with reports of nocturnal discomfort, highlights potentially important targets for future research and supportive care interventions.

With respect to HRQoL, children and adolescents with cancer reported the lowest PedsQL scores, particularly in physical, emotional, and school functioning domains, consistent with prior research. In contrast, social functioning did not differ significantly across groups. This finding may reflect relative resilience in peer relationships despite illness burden, although measurement limitations cannot be excluded and warrant further investigation.

Across the full sample, poorer sleep quality was associated with lower HRQoL, and this relationship remained significant after adjustment for demographic and clinical factors. This is consistent with previous studies demonstrating links between sleep disturbance and impaired functioning in both clinical and non-clinical paediatric populations [16,21,22]. However, inconsistent with some prior reports in oncology samples [15,32,37], within-group correlations were not statistically significant in the oncology and chronic illness groups in this study. This discrepancy may reflect limited statistical power, heterogeneity in clinical characteristics, or the influence of unmeasured factors such as treatment phase or symptom severity. Notwithstanding this, the regression findings support a broadly consistent association between sleep and HRQoL across groups.

The absence of significant differences in sleep quality by disease subtype within the oncology group suggests that sleep disturbance may represent a common burden across diagnostic categories, rather than being driven by specific malignancies. Similarly, the lack of significant gender differences is in line with some paediatric sleep literature, suggesting that clinical status may be a stronger determinant of sleep problems than sex in this context [2,3,4]. The observed age-related association with sleep quality in healthy children and adolescents, but not in clinical groups, further suggests that illness-related factors may override typical developmental influences on sleep.

## 5. Implications

Sleep disturbance emerged as a prominent and modifiable concern in paediatric oncology, particularly in domains of sleep initiation and continuity. These findings support routine integration of brief, validated sleep screening into paediatric oncology and psychosocial care pathways to enable early identification and proactive management. Positioning sleep within a biopsychosocial framework recognizes its role as a key mechanism linking treatment burden to functional and HRQoL outcomes.

Component-level results highlight actionable targets. Prolonged sleep latency may be addressed using behavioural strategies such as consistent routines, stimulus control, and management of pre-sleep arousal [38]. Sleep fragmentation and reduced efficiency appear driven by symptom burden and hospital-related disruption, supporting coordinated approaches that combine symptom optimization with sleep-promoting environments and sleep hygiene practices during treatment [33,34,35,36,39,40]. Frequent nocturnal somatic complaints further emphasize the need for multidisciplinary review of evening care routines. The exclusive use of hypnotics in oncology suggests a subgroup requiring careful monitoring and prioritization of non-pharmacological strategies. The observed association between poorer sleep and lower HRQoL suggests that sleep may represent a clinically meaningful intervention target and a useful indicator for broader psychosocial needs.

## 6. Limitations

Several limitations should be considered when interpreting these findings. First, the cross-sectional design precludes conclusions about causality or directionality in the association between sleep quality and HRQoL. It remains unclear whether sleep disturbance contributes to reduced functioning, whether poorer HRQoL adversely affects sleep, or whether both are influenced by shared underlying factors.

Secondly, participants were recruited through convenience sampling, which may have introduced selection bias and limited generalizability. Participants in the oncology and chronic illness groups were recruited from hospital settings, whereas healthy controls were recruited from the community. Differences in recruitment environments may have introduced selection bias and may limit direct comparability between groups. In particular, hospitalized children and adolescents may have experienced acute care routines, environmental disruption, or higher symptom burden at the time of questionnaire completion, whereas healthy controls completed questionnaires in community settings. Furthermore, healthy-control eligibility was based on parent- or guardian-reported health history, which may have resulted in misclassification of unrecognized or unreported conditions. Group differences should therefore be interpreted with consideration of these sampling and contextual variations.

Thirdly, sleep and HRQoL were assessed using self-report measures, which may be subject to recall bias and reporting effects. Despite the instruments being well validated, future research would benefit from incorporating objective or multi-method assessments (e.g., actigraphy, sleep diaries, or parent-proxy reports) to provide a more comprehensive evaluation of sleep patterns [41].

Fourth, the PSQI was originally developed for adults, and the Chinese PSQI has not been specifically validated across all included paediatric subgroups. Although the PSQI ≥ 5 threshold was used as a commonly applied screening cut-off for poor sleep quality, it should not be interpreted as a paediatric diagnostic criterion. Therefore, findings based on this threshold, particularly in younger children, should be interpreted cautiously.

Fifth, subgroup analyses were limited by relatively small sample sizes within specific diagnostic and sociodemographic categories, reducing statistical power to detect between-group differences. As such, non-significant findings, particularly for disease subtype comparisons, should be interpreted cautiously.

Although the total sample size was adequate for detecting the observed main group differences and the overall regression effect, the study had limited power to detect small interaction effects. The interaction block in the moderation model showed a very small effect size and low achieved power. Therefore, the non-significant moderation findings should be interpreted cautiously and should be examined in larger future studies.

In addition, several clinically relevant variables were not measured or included in the analytical models. Factors such as treatment phase, current symptom burden (e.g., pain, nausea, fatigue), psychological distress, frequency of hospitalization, and nutritional status may influence both sleep and HRQoL, and their omission may have resulted in residual confounding. Nutritional status was not systematically assessed, and standardized measures of malnutrition were unavailable. As nutritional status may influence both sleep quality and health-related quality of life, future studies should incorporate validated nutritional assessments to examine its potential contribution. Furthermore, environmental and psychological factors that may affect sleep, such as ward noise, lighting conditions, overnight clinical procedures, anxiety, stress, and emotional responses to witnessing illness or clinical deterioration in other children and adolescents, were not systematically assessed. Consequently, their contribution to the observed sleep disturbances could not be evaluated. Moreover, sleep quality was assessed at a single time point, and no follow-up data were collected after discharge. Therefore, it was not possible to determine whether the observed sleep disturbances persisted or improved following return to the home environment.

Future studies should adopt longitudinal designs to clarify temporal relationships and better delineate potential causal pathways. Incorporating detailed clinical and psychosocial variables will also be important to identify mechanisms underlying sleep disturbance and to inform targeted, developmentally appropriate interventions in paediatric oncology.

## 7. Conclusions

This study demonstrates that children and adolescents with cancer experience a higher burden of sleep disturbance than healthy peers and those with non-oncological chronic illnesses, with impairment primarily in sleep initiation and continuity rather than duration. By incorporating both chronic illness and healthy comparison groups, this study addresses a key gap by showing that sleep disturbance in paediatric oncology is not simply a feature of chronic disease but may be more severe and distinct in pattern. Future research should use longitudinal and multi-method approaches to clarify causal relationships and account for clinical and psychosocial influences. Development and evaluation of targeted, integrated sleep interventions are needed, alongside embedding routine sleep assessment into paediatric oncology care.

## Figures and Tables

**Figure 1:**
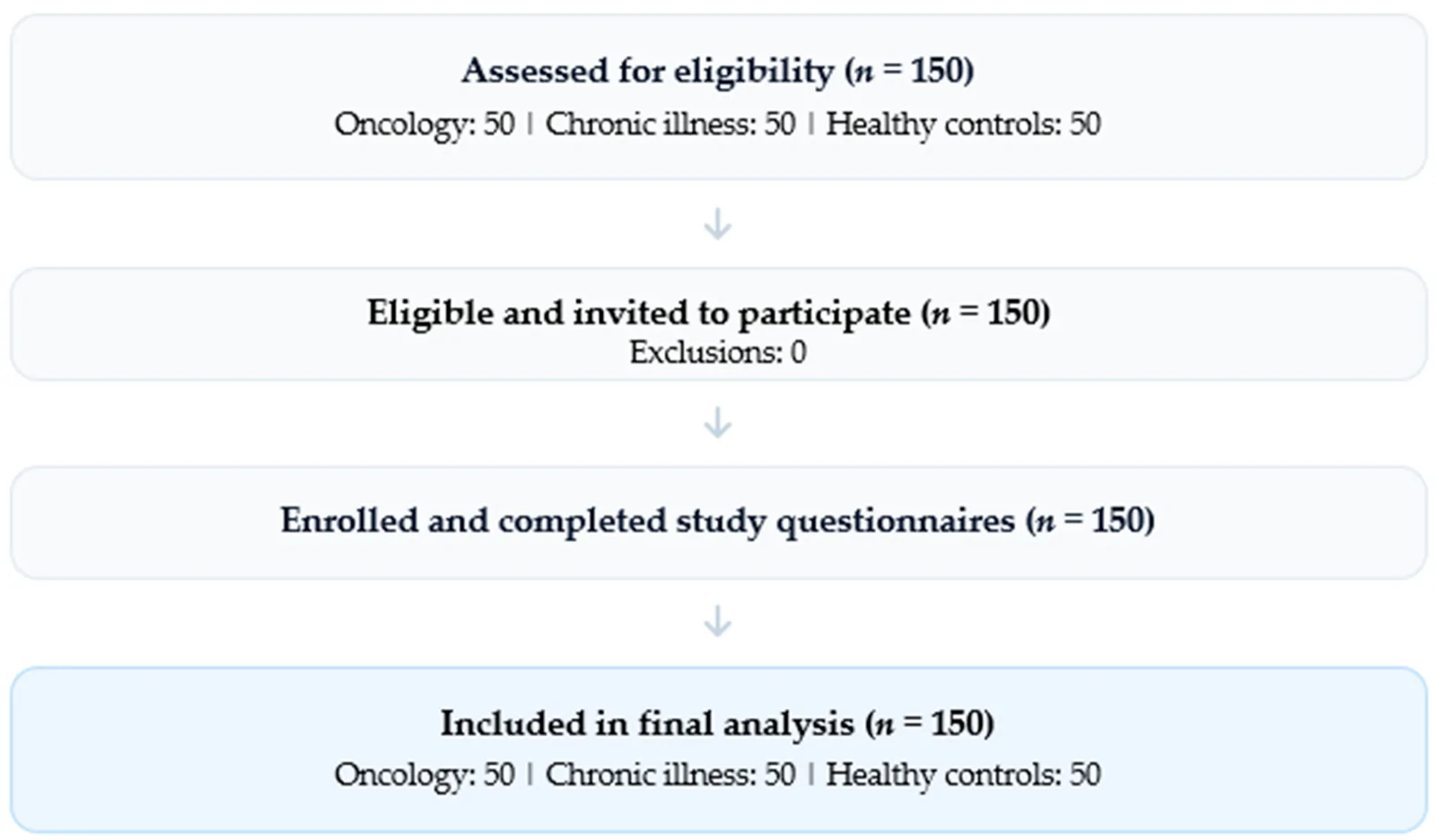
Participant Flow Diagram.

**Table 1:** Participant characteristics by group and clinical composition (disease type distribution).

Characteristic	Oncology (*n* = 50)	Chronic Illness (*n* = 50)	Healthy (*n* = 50)	F-Value/χ^2^	*p*-Value
**Age (years), mean (SD)**	11.84 (3.55)	11.80 (3.51)	11.83 (3.65)	F = 0.002	0.998
**Age group, *n* (%)**				χ^2^ = 0.856	0.652
6–12 years	25 (50.0)	25 (50.0)	29 (58.0)		
13–18 years	25 (50.0)	25 (50.0)	21 (42.0)		
**Gender, *n* (%)**				χ^2^ = 0.64	0.726
Male	25 (50.0)	27 (54.0)	23 (46.0)		
Female	25 (50.0)	23 (46.0)	27 (54.0)		
**Parent’s Education Level, *n* (%)**				χ^2^ = 18.46	0.005
Primary	6 (12.0)	2 (4.0)	0 (0.0)		
Lower secondary	8 (16.0)	9 (18.0)	3 (6.0)		
Higher secondary	11 (22.0)	20 (40.0)	12 (24.0)		
Tertiary	25 (50.0)	19 (38.0)	35 (70.0)		
**Hypnotic Use, *n* (%)**				χ^2^ = 10.35	0.006
Yes	5 (10.0)	0 (0.0)	0 (0.0)		
No	45 (90.0)	50 (100.0)	50 (100.0)		
**Disease type category, *n* (%)**					
Leukemia/Lymphoma	28 (56.0)	—	—		
Solid tumour	22 (44.0)	—	—		
Renal	—	18 (36.0)	—		
Cardiac	—	17 (34.0)	—		
Hematology/Immunology	—	5 (10.0)	—		
Neurometabolic	—	5 (10.0)	—		
Others	—	5 (10.0)	—		

**Table 2:** Pittsburgh Sleep Quality Index (PSQI) global and component scores by group.

	Oncology (*n* = 50)	Chronic Illness (*n* = 50)	Healthy (*n* = 50)	*p*-Value (3 Groups)	*p*-Value (H vs. O)	*p*-Value (H vs. CI)	*p*-Value (O vs. CI)
	Mean ± SD						
Component 1: Subjective sleep quality	1.02 ± 0.68	0.94 ± 0.87	0.66 ± 0.63	0.039	0.040	0.14	0.85
Component 2: Sleep latency	1.56 ± 0.91	0.90 ± 0.84	0.76 ± 0.80	<0.001	<0.001	0.69	<0.001
Component 3: Sleep duration	0.34 ± 0.75	0.26 ±0.63	0.26 ± 0.49	0.77	0.80	1.00	0.80
Component 4: Habitual sleep efficiency	0.66 ± 0.98	0.26 ±0.66	0.12 ± 0.48	0.001	0.001	0.61	0.021
Component 5: Sleep disturbances	1.26 ± 0.66	1.10 ± 0.46	0.78 ± 0.46	<0.001	<0.001	0.010	0.30
Component 6: Use of sleeping medication	0.18 ± 0.63	0.00 ±0.00	0.00 ± 0.00	0.019	0.038	1.00	0.038
Component 7: Daytime dysfunction	0.94 ± 0.68	0.64 ±.60	0.72 ± 0.70	0.067	0.22	0.82	0.064
Global PSQI score	6.02 ± 3.40	4.10 ±2.60	3.30 ± 2.35	<0.001	<0.001	0.33	0.002

O: Oncology group; CI: Chronic Illness group; H: Healthy group; One-way ANOVA for overall comparison; Tukey’s HSD post hoc test for pairwise comparison. Values are presented as mean +/− standard deviation and represent original, uncentered PSQI scores.

**Table 3:** Pediatrics Quality of Life Inventory (PedsQL) scores by group.

	Oncology (*n* = 50)	Chronic Illness (*n* = 50)	Healthy (*n* = 50)	*p*-Value (3 Groups)	*p*-Value (H vs. O)	*p*-Value (H vs. CI)	*p*-Value (O vs. CI)
	Mean ± SD						
Physical Functioning score	68.14 ± 22.41	79.95 ± 19.37	92.88 ± 8.88	<0.001	<0.001	<0.001	0.006
Emotional Functioning score	66.00 ± 22.13	77.40 ± 20.13	80.70 ± 17.23	<0.001	<0.001	0.38	0.008
Social Functioning score	82.40 ± 13.93	82.90 ± 17.20	86.80 ± 15.14	0.30	0.13	0.23	0.87
School Functioning score	59.80 ± 20.23	63.60 ± 16.32	77.00 ± 16.75	<0.001	<0.001	<0.001	0.30
Psychosocial Health Summary Score	68.87 ± 15.36	75.10 ± 14.45	81.44 ± 12.85	<0.001	<0.001	0.023	0.039
Global PedsQL score	68.98 ± 15.73	75.99 ± 14.03	85.44 ± 10.26	<0.001	<0.001	<0.001	0.021

O: Oncology group; CI: Chronic Illness group; H: Healthy group; One-way ANOVA for overall comparison; Tukey’s HSD post hoc test for pairwise comparison.

**Table 4:** PSQI-PedsQL correlations by group.

Sample	Pearson r	*p*-Value
Total sample	−0.378	<0.001
Oncology group	−0.184	0.201
Chronic Illness group	−0.253	0.076
Healthy group	−0.454	0.001

**Table 5:** Multiple linear regression of PedsQL total score: Model 1 without interaction terms.

Independent Variables	B (SE)	β	*p*
**Mean-centered global PSQI**	−1.347 (0.436)	−0.271	0.002
**Age (years)**	0.106 (0.325)	0.025	0.744
**Study group**			
Healthy (reference)			
Oncology	−13.510 (2.935)	−0.425	<0.001
Chronic Illness	−8.485 (2.772)	−0.267	0.003
**Gender**			
Male (reference)			
Female	−0.807 (2.254)	−0.027	0.721
**Use of hypnotics**			
Yes (reference)			
No	−6.860 (6.747)	−0.082	0.311
**Parental Education**			
Tertiary (reference)			
Primary	−2.054 (5.542)	−0.031	0.711
Lower secondary	2.240 (3.458)	0.051	0.518
Higher secondary	−0.811 (2.639)	−0.024	0.759

Note: B = unstandardized regression coefficient; SE = standard error; β = standardized regression coefficient. The healthy group was the reference group. R^2^ = 0.268, Adj. R^2^ = 0.221, F(9,140) = 5.706, *p* < 0.001.

**Table 6:** Multiple linear regression of PedsQL total score: Model 2 including PSQI × group interactions.

Independent Variables	B (SE)	β	*p*
***Mean-centered global PSQI***	−2.246 (0.860)	−0.452	0.010
***Age (years)***	0.240 (0.343)	0.057	0.485
***Study group***			
Healthy (reference)			
Oncology	−12.767 (3.056)	−0.401	<0.001
Chronic Illness	−7.236 (2.983)	−0.227	0.017
***Gender***			
Male (reference)			
Female	−1.231 (2.283)	−0.041	0.591
***Use of hypnotics***			
Yes (reference)			
No	−4.344 (7.133)	−0.052	0.544
***Parental Education***			
Tertiary (reference)			
Primary	−4.198 (5.798)	−0.063	0.470
Lower secondary	1.887 (3.477)	0.043	0.588
Higher secondary	−1.195 (2.660)	−0.036	0.654
**Interactions**			
Mean-centered global PSQI × oncology indicator	1.433 (1.115)	0.198	0.201
Mean-centered global PSQI × chronic illness indicator	0.999 (1.145)	0.100	0.384

Note: B = unstandardized regression coefficient; SE = standard error; β = standardized regression coefficient. The healthy group was the reference group. Model 2 includes all Model 1 predictors plus two interaction terms. R^2^ = 0.277, Adj. R^2^ = 0.219, F(11,138) = 4.808, *p* < 0.001. Interaction block: ΔR^2^ = 0.009; ΔF(2,138) = 0.829, *p* = 0.438. The PSQI coefficient represents the slope in the healthy reference group; interaction coefficients represent differences in slopes relative to that group. Group coefficients represent adjusted differences in PedsQL total score at a centered PSQI score of zero, corresponding to an original PSQI score of approximately 4.47. This interpretation follows the coding of continuous-by-categorical interactions.

## Data Availability

The data that support the findings of this study are available from the corresponding author upon reasonable request. Data may not be publicly shared due to privacy and ethical restrictions that protect participant confidentiality.

## Supplementary Materials

The following supporting information can be downloaded at https://www.mdpi.com/article/10.3390/diseases14100356/s1, Table S1: PSQI scores by group using Kruskal–Wallis tests; Table S2: Comparison of original ANOVA Table 2 vs. Kruskal–Wallis output.

## Abbreviations

The following abbreviations are used in this manuscript:

HRQoL	Health-related Quality of Life
HKCH	Hong Kong Children’s Hospital
PSQI	Pittsburgh Sleep Quality Index
PedsQL 4.0	Pediatrics Quality of Life Inventory 4.0
