# Supplementary material for: Sleep Disturbances and Health-Related Quality of Life in Paediatric Oncology: A Comparative Study with Non-Oncological Chronic Illness and Healthy Peers
Source: Diseases. 2026 Sep 26;14(10):356. doi: 10.3390/diseases14100356 (PMC13703032; doi:10.3390/diseases14100356)
Supplement: Supplementary file 1 [file diseases-14-00356-s001.zip › diseases-4450801-supplementary.pdf]

Table S1: **PSQI scores by group using Kruskal–Wallis tests**

**PSQI variable**	**Oncology (*n*=50)**<br>**Mean ± SD**	**Chronic illness (*n*=50)**<br>**Mean ± SD**	**Healthy (*n*=50)**<br>**Mean ± SD**	**Kruskal–Wallis H**	**df**	***p*-value**	**ε^2**
Component 1: Subjective sleep quality	1.02 ± 0.68	0.94 ± 0.87	0.66 ± 0.63	7.219	2	0.027	0.036
Component 2: Sleep latency	1.56 ± 0.91	0.90 ± 0.84	0.76 ± 0.80	21.763	2	< 0.001	0.134
Component 3: Sleep duration	0.34 ± 0.75	0.26 ± 0.63	0.26 ± 0.49	0.406	2	0.816	—
Component 4: Habitual sleep efficiency	0.66 ± 0.98	0.26 ± 0.66	0.12 ± 0.48	14.598	2	< 0.001	0.086
Component 5: Sleep disturbances	1.26 ± 0.66	1.10 ± 0.46	0.78 ± 0.46	18.196	2	< 0.001	0.110
Component 6: Use of sleeping medication	0.18 ± 0.63	0.00 ± 0.00	0.00 ± 0.00	10.273	2	0.006	0.056
Component 7: Daytime dysfunction	0.94 ± 0.68	0.64 ± 0.60	0.72 ± 0.70	5.295	2	0.071	0.022
Global PSQI score	6.02 ± 3.40	4.10 ± 2.60	3.30 ± 2.35	21.857	2	< .001	0.135

Table S2: **Comparison of original ANOVA Table 2 vs. Kruskal–Wallis output**

**PSQI component**	**Original ANOVA *p*-value**	**ANOVA conclusion**	**Kruskal–Wallis *p*-value**	**Kruskal–Wallis conclusion**	**Match?**
Component 1: Subjective sleep quality	0.039	Significant	0.027	Significant	Yes
Component 2: Sleep latency	< 0.001	Significant	< 0.001	Significant	Yes
Component 3: Sleep duration	0.770	Not significant	0.816	Not significant	Yes
Component 4: Habitual sleep efficiency	0.001	Significant	< 0.001	Significant	Yes
Component 5: Sleep disturbances	< 0.001	Significant	< 0.001	Significant	Yes
Component 6: Use of sleeping medication	0.019	Significant	0.006	Significant	Yes
Component 7: Daytime dysfunction	0.067	Not significant	0.071	Not significant	Yes

Kruskal–Wallis sensitivity analyses showed a similar pattern of findings to the one-way ANOVA results and did not change the interpretation of the PSQI component comparisons.
